# The feasibility of the ACOSOG Z0011 Criteria to Chinese Breast Cancer Patients: A Multicenter Study

**DOI:** 10.1038/srep15241

**Published:** 2015-10-16

**Authors:** Miao Liu, Shu Wang, Shude Cui, Xuening Duan, Zhimin Fan, Zhigang Yu

**Affiliations:** 1 Breast Center, Peking University People’s Hospital, Beijing, China; 2 Department of Breast Surgery, Cancer Center of Henan Province, China; 3 Breast Disease’s Center, The first Affiliated Hospital of Peking University, China; 4 Department of Breast Surgery, The First Affiliated Hospital of Jilin Univesity, China; 5 Department of Breast Surgery, The Second Affiliated Hospital of Shandong University, China

## Abstract

The aim of this study was to determine the feasibility of the Z0011 criteria to Chinese breast cancer patients. An survey about the Z0011 trial was distributed and we collected 658 consecutive patients with axillary lymph node dissection (ALND) after positive sentinel lymph node (SLN) biopsy from five centers’ databases and grouped them as eligible or ineligible for omitting ALND according to the Z0011 criteria. The eligible group was compared with the cohort included in the Z0011 trial and with the ineligible group. Of the 427 respondants, 106 (24.8%) and 130 (30.4%)would not routinely perform ALND in patients meeting Z0011 criteria before and after learning of the trial results, respectively. Among the 658 patients, 151 (22.9%) were eligible and 507 were ineligible for omitting ALND. The clinicopathologic factors were not statistically different between the eligible group and the Z0011 cohort. Compared with the eligible Group, the ineligible group had significantly more T2 and T3 stage tumors, positive lymph nodes(LNs) and positive non-sentinel lymph nodes (NSLNs) (P < 0.01). The findings suggest good exportability of the Z0011 criteria to Chinese patients omitting ALND, but application of Z0011 as national treatment guideline still needs additional time and effort.

Sentinel lymph node (SLN) biopsy is currently the standard of care for staging clinically negative axilla in breast cancer patients, with axillary lymph node dissection(ALND) reserved for patients with metastases found by SLN biopsy^1^,^2^,^3^. The American College of Surgeons (ACOSOG) Z0011 trial demonstrated that in clinically node -negative women undergoing breast-conserving therapy (BCT) and found to have metastases to 1 or 2 SLNs, sentinel lymph node biopsy (SLNB) alone resulted in rates of local control, disease-free survival (DFS), and overall survival (OS) equivalent to those seen after ALND but with significantly lower morbidity^4^,^5^. Based on their findings, current guidelines from the American Society of Clinical Oncology and the National Comprehensive Cancer Network recommend considering no further surgery for patients who meet ACOSOG Z0011 eligibility criteria^6^.

Several surveys conducted by researchers in the United States revealed that many surgeons were likely to incorporate Z0011 into their practice^7^,^8^ and several centers have already modified their practice based on the Z0011 criteria^9^,^10^. Practice in China has been slower to incorporate the Z0011 criteria into its standard of care for some reasons. It is well known that there are some differences in the clinical characteristics between Chinese breast cancers patients and westerners. One of our previous studies has shown that western non-sentinel lymph node (NSLN) metastasis predicting nomogram dose not perform as well as in their original studies when used in Chinese patients^11^. This is a strong evidence to support that Chinese breast cancer patients are different from western populations. Therefore questions have been raised about whether data analyzing a US cohort in Z0011 are applicable to Chinese patients. Moreover there is no current information regarding how Z0011 has affected Chinese surgeons’ practice patterns across the nation.

So the purpose of this study is to assess the impact of Z0011 on surgeons’ practices across China, analyse the clinical relevance of the Z0011 findings for Chinese breast cancer patients and eventually evaluate the feasibility of using the Z0011 criteria to omit axillary lymph node dissection after positive sentinel lymph node biopsy in Chinese breast cancer patients.

## Methods

### Survey

The survey designed as detailed below was reviewed by the Chinese Preventive Medicine Association Research Committee and approved by the Board of Directors.

A seven-question survey was sent to every member of Surgical Group of Breast Cancer affiliated with Chinese Preventive Medicine Association by electronic mail. Five questions assessed the grade of the hospital in which each surgeon practices; the location of each surgeon’s affiliation; and each surgeon’s degree, practice duration and familiarity with Z0011. The sixth question assessed preferences for treatment of patients who meet the inclusion criteria and the treatment protocol of the Z0011 trial: T1 or T2, clinically node-negative invasive breast cancer ; one or two positive SLNs by routine haematoxylin and eosin (H&E) staining or frozen section; and treatment with breast-conserving therapy(BCT), whole breast irradiation(WBI), and adjuvant systemic therapy (chemotherapyand/or endocrine therapy) before learning of Z0011. After a simple explanation of Z0011’s results, the last question assessed the impact of Z0011 on the management of the same patients as the sixth question after the surgeon had learned of Z0011.

The first e-mail with request to complete the survey was sent on August 1, 2014 and the survey was closed on October 1, 2014.

### Study patients

We reviewed the medical records of 658 breast cancer patients admitted to five different hospitals from December 2008 through October 2014. 194 patients from the Breast Center of Peking University People’s Hospital(PKUPH )(Beijing, China), 206 patients from Breast Surgery Service of Cancer Center of Henan Province(Zhengzhou, Beijing), 161 patients from Breast Disease Center of First Hospital of Peking University (Beijing, China), 66 patients from Breast Surgery Service of First Hospital of Jilin University(Changchun, China), and 31 patients from Breast Surgery Service of Second Hospital of Shandong University(Jinan, China). Patients were selected if they had pathologically confirmed breast cancer with clinically negative lymph nodes before surgery, had positive SLNs on routine H&E staining or frozen section, had not received any neoadjuvant systemic therapy and had ALND after positive sentinel lymph node biopsy(SLNB). Approval from Peking University People’s Hospital’s review board was obtained before data collection. The patient data collection methods were carried out in accordance with the approved guidelines. Written consent was obtained from all the patients.

### Surgery and SLN pathological evaluation

SLNs were identified using fluorescence and/or blue dye according to surgeon preference. Intraoperative frozen section was performed on all SLNs. The SLN was cut longitudinally into 2 halves. Half of the node was frozen for immediate examination, and up to 2 sections were stained with H&E. The other half node was fixed in formalin and embedded in paraffin, and up to 2 sections were stained with H&E. Immunohistochemical stain was not routinely used in the diagnosis of SLN metastasis.

Axillary dissection was performed if SLN was positive by frozen section analysis. Patients with SLN metastases that were not detected during surgery generally underwent completion ALND at a later date. For all additional nodes identified by completion ALND, routine H&E analysis was conducted on a single section of each node.

### Clinical and pathological characters

The clinical and pathological data collected for each case included age, clinical tumor size, nuclear grade, estrogen receptor (ER) expression, progesterone receptor (PR) expression, number of positive SLN, number of positive LN, number of positive non-sentinel lymph node (NSLN), surgical treatment (breast conserving therapy (BCT) or mastectomy), adjuvant systemic chemotherapy and/or endocrine therapy after surgery (yes or no) and whole breast irradiation after surgery(yes or no).

### Comparison and Statistical Analysis

The survey results were analysed with descriptive statistics.

Factors associated with surgeons’ surgical practice pattern were analysed using Chi-squared test and Fisher’s exact test.

All the 658 patients were divided into an eligible group comprising patients who met the Z0011 criteria for omitting ALND and an ineligible group comprising patients who did not meet these criteria. The eligible group was compared with the cohort included in the “ALND” arm of the Z0011 trial (n = 420, in intention to treat) and the eligible group was also compared to the ineligible group. Chi-squared test and Fisher’s exact test were used for categorical variables.

SPSS19.0 software was used for statistical analyses. A *P* value of <0.05 was considered significant.

## Results

427 surgeons completed the online questionnaire. The information in the survey is listed in Table 1. The majority (75.9%) of surgeons practiced in a third-grade class-A hospital, which is the highest level granted to a hospital according to Chinese health administrative department provision. 283(66.3%) surgeons practiced in municipalities directly under central government. A total of 185(43.3%) had medical master degrees and 122(28.6%) had acquired doctor degrees. Practice duration varied among the respondents : 120(28.1%) had been in practice for 0–5 years, 168(39.3%) for 5–10 years, 101(23.7%) for 10–15 years, and 38(8.9%) for over 20 years. Only 116(27.2%) of respondents indicated familiarity with Z0011. Before learning of the trial results, 144(33.7%) would perform completion ALND all of the time on a woman eligible by the Z0011 criteria, 177(41.5%) would perform completion ALND most of the time, and 106(24.8%) would not routinely perform ALND. Whether or not the surgeon was aware of Z0011, we explained the Z0011 trial results in our survey. After learning of them, 119 (27.9%) would perform completion ALND all of the time, 178(41.7%) would perform completion ALND most of the time, and 130(30.4%) would not routinely perform ALND in such patients.

According to the respondents’ opinions of surgical treatment for patients fulfilling the criteria of Z0011 after learning of the trial results, we divided them into one group of ALND preference including surgeons who would perform completion ALND all of the time or most of the time and another group of ALND non-preference, including those who would not routinely perform ALND. The statistical result indicated there were no correlations between grade of hospital, location of hospital, surgeon’s degree, surgeon’s practice duration and surgeon’s preference for ALND after they learned of the Z0011 results (*p* < 0.05) (Table 2).

**Figure 1:**
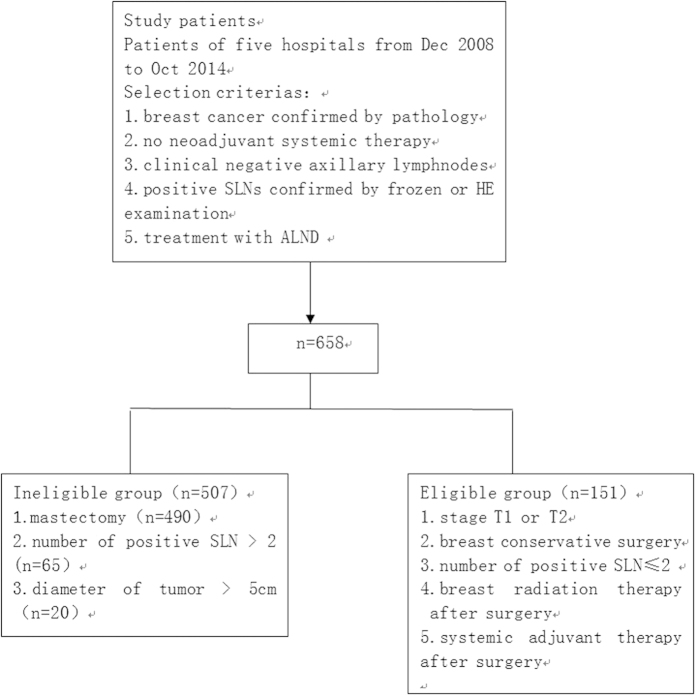
Diagram of patient groups.

Among the 658 patients enrolled in the current study, 151(22.9%) fulfilled the Z0011 criteria: clinical stage T1 or T2 breast cancer; one or two positive SLNs; and treatment with breast-conserving therapy, whole breast irradiation, and adjuvant systemic therapy (chemotherapy and/or endocrine therapy) and were assigned to the eligible group. The rest of the patients (507, 77.1%) were assigned to the ineligible group. In the ineligible group, 490 patients had a mastectomy, 65 patients had more than 2 positive SLNs, and 20 patients had tumors with diameter more than 5 cm (Fig. 1).

The ER status, clinical stage, number of positive lymph nodes (LNs) and positive NSLNs were not significantly different between the eligible group and the Z0011 cohort. The patients in the eligible group had more grade II tumors than the Z0011 cohort (p = 0.001) (Table 3).

Compared with the eligible group in this study, the ineligible group had significantly more T2 and T3 stage tumors, positive LNs and positive NSLNs (P < 0.01). However there was no significant difference in ER status, clinical stage and nuclear grade between these two groups (Table 4).

## Discussion

Prior to publication of Z0011, it had been questioned whether ALND could be omitted in selected patients with a positive SLNB. Several retrospective studies^12^,^13^,^14^,^15^,^16^ have shown no significant difference in locoregional recurrence rates in patients with positive SLNs who had SLNB only compared with those who underwent ALND. There have also been prospective non-randomized studies^17^,^18^ showing similar results; however these patient cohorts were small and follow-up was less than five years.

The ACOSOG Z0011 trial is the largest prospective randomized trial thus far, and it compares locoregional recurrence rates and survival in women with positive sentinel nodes treated with SLNB or ALND. This trial defined a select cohort of patients in whom completion ALND may be safely omitted as long as the patients fulfill the criteria of clinical stage T1 or T2, N0, M0 breast cancer; one or two positive SLNs; treatment with breast-conserving therapy, whole breast irradiation, and adjuvant systemic therapy (chemotherapy and/or endocrine therapy).

During the past five years since the publication of Z0011, the study has been described as a practice-changing trial^19^. Some centers in the United States, Europe and Australia have applied Z0011 criteria to their own population and found the criteria is eligible^10^,^20^,^21^,^22^. However, in China ALND is still recommended for patients with positive SLNs according to the guideline of Breast Cancer Committee affiliated with Chinese Anti-Cancer Association^23^. The current study aimed to investigate the feasibility of applying the Z0011 criteria in China. To our knowledge, no other studies have addressed this question before.

It is well known that the feasibility of new practice protocol in China depends on two aspects: the subjective opinions from clinicians regarding the protocol and the clinical applicability of the protocol to Chinese patients. Therefore, our study included two parts: a survey assessing the impact of Z0011 on surgeons’ practices across China, and a multi-center study analysing the clinical relevance of the Z0011 findings to Chinese breast cancer patients.

Among the 427 respondents to the survey, most surgeons came from third-grade class-A hospital (75.9%) and municipalities or provincial capitals (87.4%). In China, the training system after graduation is for clinicians from community hospitals to receive training in hospitals of high grade. We believe the answers of the 427 surgeons could represent the current pattern of breast surgery practice in our country.

Although 71.9% of the respondents had a postgraduate education background, it was disappointing that only 27.2% indicated familiarity with Z0011 trial. In a survey given to members of the American Society of Breast Surgeons (ASBrS), 97% of 849 respondents were familiar with Z0011^8^. Another questionnaire survey distributed to members of the North Pacific Surgical Association and the Oregon and Washington chapters of the American College of Surgeons found a similar result, with 94% of surgeons being aware of Z0011^24^.

Our survey data still indicated before Z0011’s publication, 75.2% of the respondents would perform ALND most or all of the time on patients meeting the Z0011 criteria. In western countries, some surgeons had elected to omit ALND in some patients with a positive SLN or favorable clinicopathologic characteristics prior to publication of Z0011^15^,^18^,^25^,^26^. Yi *et al.* published data from the Surveillance, Epidemiology, and End Results (SEER) database on 26,986 breast cancer patients with nodal disease treated from 1998 to 2004. Completion ALND was omitted in 16.4%^13^. After Z0011’s publication, many studies indicated the majority of surgeons incorporated Z0011 into practice by omitting completion ALND in patients with one or two positive SLNs undergoing BCT who were targeted for WBI^7^,^8^,^10^,^13^. Gainer *et al.*’s survey results showed that 468 (56.9%) respondents would infrequently or never perform completion ALND on a woman fulfilling Z0011 criteria, while 186 (22.6%) would sometimes perform completion ALND and 168 (20.4%) would perform completion ALND most or all of the time^8^. By contrast, our current survey data demonstrated after learning of Z0011 results from our explanation shown in the questionnaire, 69.6% of the respondnts would still perform ALND most or all of the time on patients meeting the Z0011 criteria.

It was interesting that the result of our survey demonstrated that there was no difference in the physicians’ preference for ALND after learning of Z0011, regardless of whether the physician practiced in a high-level hospital or a lower one, came from a metropolis or a small town, had a doctoral degree or only a bachelor degree, or had practiced for a longer time or a shorter time.

It seemed that the Z0011 trial did not have the same impact on Chinese surgeons’ practice as on western surgeons’^8^,^24^. Even having learned of the Z0011 results, most Chinese surgeons would much more frequently choose radical surgery for patients with positive SLNs. Although lack of detailed knowledge regarding Z0011 might affect respondents’ acceptance of Z0011 to some extent in the current study, we thought that the survey was consistent with the current management pattern for breast cancer patients with positive SLNs in China.

Although we did not survey in detail the reasons why Chinese physicians would not alter their practice mode according to Z0011 results, we could presume the main cause is because Z0011 was recently the hot topic of discussion at many breast cancer meetings in China . First, most Chinese surgeons worry about the recurrence of breast cancer if positive lymphnodes are left in the axilla. The completeness of tumour resection has been the standard for evaluating the success of surgery in our practice, and recurrence at local or regional nodes would be attributed to incomplete excision. Second, in many areas of China, especially suburban areas, many patients could not undergo standard adjuvant treatment after surgery due to many reasons, including medical conditions, patient intention, economic ability, etc. Thus, many Chinese physicians would argue that the Z0011 trial does not fit China’s basic conditions. Third, there are differences in clinical characteristics between Chinese breast cancer patients and Westerners, and no data about the relevance of the Z0011 criteria to Chinese patients have been reported until now. It is reasonable that Chinese physicians are uncertain as to whether western trial results are applicable to the Chinese population.

To address the third point above, we collected 658 consecutive breast cancer patients from five hospitals’ databases to examine whether following the Z0011 criteria would select patients with the same characteristics as those of the cohort included in the Z0011 trial.

At these five hospitals ALND was the standard treatment for patients with positive SLN regardless of the size of metastasis or number of positive SLNs. After applying Z0011 criteria to the 658 patients with ALND after positive SLNB, we found 151(22.9%) patients were eligible for omitting ALND. Yi *et al.* found 36.6% of patients with positive SLNs could have SLNB only using Z0011 criteria^27^. Delpech *et al.* found 69.6% of patients with positive SLNs met Z0011 criteria^20^. Authors from Memorial Sloan-Kettering Cancer Center found that 48% of patients with positive SLNB would be spared ALND if they incorporate Z0011 results into their cancer care^28^. The difference between our study and western ones might be mainly due to the lower rate of BCT^20^,^27^,^29^. In our study, 490(74.5%) patients had mastectomies and 445(67.6%) patients could not be spared ALND only because they underwent mastectomy, according to the Z0011 results.

The rate of BCT in China varies greatly among different areas and hospitals. After more than twenty years’ practice, BCT has been widly accepted across China and has become the most common surgery for breast cancer. The five hospitals attending this study could represent the highest level for breast surgery in China, but the rate of BCT in this study is lower than in other studies in western countries. The difference of BCT may mainly because of the difference of medical conditions and economic ability in China and western countries. On the other hand, Chinese patients usually having smaller size breasts than westerners may be another cause. But since the development of medical technique and economy in China, the rate of BCT would be higher in the future. Therefore, discussing the Z0011 trial’s applicability to Chinese patients will be more meaningful.

Compared with axillary clearance, SLNB is associated with significantly less morbidity, such as seroma formation, wound infection, arm dysfunction, sensory loss and lymphoedema^30^,^31^,^32^,^33^,^34^. So if using Z0011 findings to Chinese breast cancer patients with metastatic SLNs could make nearly one quarter of them avoiding ALND safely. This result suggests that applying Z0011 criteria to Chinese patients has great clinical meaning.

We found no significant difference in clinical characteristics including ER status, clinical stage, or number of positive LNs or positive NSLNs between our group of patients who were potentially eligible for omitting ALND according to the Z0011 criteria and the population reported in the Z0011 trial. The patients in the eligible group had more gradeII tumors than the Z0011 cohort (p = 0.001), perhaps because we classified all gradeI and gradeII mixed tumors as gradeII tumors all. This finding indicated using Z0011 results in Chinese patients could select ones with similar characteristics as the Z0011 cohort, which supports the safe exportability of the Z0011 criteria. In our comparison of the patients who were ineligible for omitting ALND with those who were eligible, the ineligible group had a significantly higher percentage of patients with later stage tumors and advanced disease in the axilla. This result demonstrated that the Z0011 criteria might discriminate patients with a high risk of recurrence and metastasis from those with a low risk, and it confirms the importance of the strict adherence to the Z0011criteria in China.

However, there has been some criticism on Z0011^35^,^36^. The trial had a potential bias related to the enrolment of only patients with a good prognosis, and it was closed early with less than 50% target accrual. There were also high survival and low event rates (only 92 events for the trial), and a large number of women in both groups were lost to follow up—21% in the axillary dissection group and 17% in the SLNB group. Lastly, the radiation techniques were not described, including dosing, frequency and field definitions. While many centers have changed their treatment pattern according to Z0011 results and acquired similar survival rate as Z0011 trial^10^,^20^,^27^. It is important that future trials should focus on groups excluded from Z0011, such as patients undergoing neoadjuvant chemotherapy or neoadjuvant hormonal therapy, mastectomy, or radiation modalities other than WBI^37^.

The major limitation of our study is that we could only collect retrospective data because ALND is the standard treatment for patients with positive SLNs. The results of our study indicat good applicability of Z0011criteria in Chinese Patients, and we need further data on the application of the Z0011 criteria in China.

From the results of our survey, we conclude that time is to be needed before the Z0011 criteria becomes the standard guideline in China. The same as the acceptance of BCT and SLNB in China, it will require a process of development. We should first begin to incorporate Z0011 into our practice in hospitals with suitable medical conditions, such as the five hospitals participating in this study. The five hospitals have played a leading role in the breast surgery field in China, and we believe that changes in practice at these five hospitals would have a strong impact on others. In addition, more introduction and explanation of Z0011 trial to more Chinese physicians through meeting, press, and the Internet would help to make it more acceptable in China.

We think the results of our current multicenter study offer data regarding our own population and may influence Chinese surgeons to incorporate the Z0011 criteria into their practice.

## Additional Information

**How to cite this article**: Liu, M. *et al.* The feasibility of the ACOSOG Z0011 Criteria to Chinese Breast Cancer Patients: A Multicenter Study. *Sci. Rep.* **5**, 15241; doi: 10.1038/srep15241 (2015).

## Figures and Tables

**Table 1:** Survey information.

**question**	**n**	**%**
Grade of hospital		
Third-grade class-A hospital	324	75.9
Third-grade class-B hospital	58	13.6
Second-grade hospital	45	10.5
Location of affiliation		
municipality	283	66.3
Provincial capital	90	21.1
Prefecture-level city	36	8.4
County-level city	14	3.3
County/town	4	0.9
Degree		
Bachelor	110	25.8
Master	185	43.3
Doctor	122	28.6
Junior college	10	2.3
Practice duration		
0–5 years	120	28.1
6–10 years	168	39.3
11–15 years	101	23.7
over 20 years	38	8.9
Familiarity with Z0011		
Familiar with	116	27.2
Heard of but not familiar with	269	63.0
Never heard of	42	9.8
Practice pattern before learning of Z0011 trial results		
perform completion ALND all of the time	144	33.7
perform completion ALND most of the time	177	41.5
would not routinely perform ALND	106	24.8
Practice pattern after learning of Z0011 trial results		
perform completion ALND all of the time	119	27.9
perform completion ALND most of the time	178	41.7
would not routinely perform ALND	130	30.4

**Table 2:** Factors associated with surgeons’ acceptance of Z0011.

**Factors**	ALND preference*		**ALND non-preference**		***p***
	**n = 297**		**n = 130**		
	**n**	**%**	**n**	**%**	
Grade of hospital					
Third-grade class-A hospital	224	75.4	100	76.9	0.385
Third-grade class-B hospital	38	12.8	20	15.4	
Second-grade hospital	35	11.8	10	7.7	
Location of affiliation					
municipality	203	68.4	80	61.5	0.376
Provincial capital	58	19.5	32	24.6	
Prefecture-level city and inferior districts	36	12.1	18	13.8	
Degree					
Doctor	85	28.6	37	28.5	0.804
Master	126	42.4	59	45.4	
Bachelor and inferior	86	29.0	34	26.2	
Practice duration					
0–5 years	84	28.3	36	27.7	0.597
6–10 years	120	40.4	48	36.9	
11–20 years	65	21.9	36	27.7	
over 20 years	28	9.4	10	7.7	

**Table 3:** The clinical characteristics of patients eligible for omitting ALND according to ACOSOG Z0011 criteria in the current study and in the ALND arm of the Z0011 trial.

**Clinicopathologic characteristics**	**Current study eligible group**		**Z0011 ALND arm**		***p***
	**n = 151**		**n = 420**		
	**n**	**%**	**n**	**%**	
Median age	47		51		
Clinical T stage					0.925
T1	100	66.2	284	67.6	
T2	50	33.1	134	31.9	
Missing	1	0.7	2	0.5	
Median clinical tumor size(cm)	1.6		1.7		
ER					0.859
Positive	124	82.1	317	75.5	
Negative	27	17.9	66	15.7	
Missing	0	0	37	8.8	
Nuclear grade					0.001
I	10	6.6	71	16.9	
II	84	55.6	158	37.6	
III	35	23.2	94	22.4	
Missing	22	14.6	97	23.1	
Positive LN					0.431
0	0	0	4	1	
1	91	60.3	199	47	
2	35	23.2	68	16	
≥3	25	16.6	72	17	
Missing	0	0	77	18	
NSLN status					0.968
Positive	38	25.2	97	23	
Negative	113	74.8	291	69	
Missing	0	0	32	8	

**Table 4:** Clinical characteristics of patients in the current study eligible for omitting ALND and ineligible for omitting ALND according to the ACOSOG Z0011 criteria.

**Clinicopathologic characteristics**	**Current study SLND arm**		**Current study ALND arm**		***p***
	**n = 151**		**n = 507**		
	**n**	**%**	**n**	**%**	
Median age	49		51		
Clinical T stage					0.000
T1	100	66.2	248	48.9	
T2	50	33.1	239	47.1	
T3	0	0	20	3.9	
Missing	1	0.7	0	0	
Median clinical tumor size(cm)	1.6		2.2		
ER					0.620
Positive	124	82.1	425	83.8	
Negative	27	17.9	82	16.2	
Missing	0	0	0	0	
Nuclear grade					0.09
I	10	6.6	41	10.1	
II	84	55.6	240	47.3	
III	35	23.2	65	12.8	
Missing	22	14.6	161	31.8	
Positive LN					0.000
0	0	0	0	0	
1	91	60.3	233	46.0	
2	35	23.2	105	20.7	
≥3	25	16.6	169	33.3	
Missing	0	0	0	0	
NSLN status					0.005
Positive	38	25.2	191	37.7	
Negative	113	74.8	316	62.3	
Missing	0	0	0	0	
